# Optofluidic lens with tunable focal length and asphericity

**DOI:** 10.1038/srep06378

**Published:** 2014-09-16

**Authors:** Kartikeya Mishra, Chandrashekhar Murade, Bruno Carreel, Ivo Roghair, Jung Min Oh, Gor Manukyan, Dirk van den Ende, Frieder Mugele

**Affiliations:** 1 University of Twente – MESA+ institute for Nanotechnology – Physics of Complex Fluids; PO Box 217; 7500 AE Enschede (The Netherlands); 2 Ivo Roghair Eindhoven University of Technology, Department of Applied Physics, Mesoscopic Transport Properties GroupP.O. Box 513, 5600MB Eindhoven (The Netherlands)

## Abstract

Adaptive micro-lenses enable the design of very compact optical systems with tunable imaging properties. Conventional adaptive micro-lenses suffer from substantial spherical aberration that compromises the optical performance of the system. Here, we introduce a novel concept of liquid micro-lenses with superior imaging performance that allows for simultaneous and independent tuning of both focal length and asphericity. This is achieved by varying both hydrostatic pressures and electric fields to control the shape of the refracting interface between an electrically conductive lens fluid and a non-conductive ambient fluid. Continuous variation from spherical interfaces at zero electric field to hyperbolic ones with variable ellipticity for finite fields gives access to lenses with positive, zero, and negative spherical aberration (while the focal length can be tuned via the hydrostatic pressure).

Compactness and optical performance are frequently competing design criteria for high tech micro-optical imaging systems^1^ in various application areas including consumer electronics, medical devices, and military equipment. When spatial constraints prevent the use of complex compound optical systems adaptive singlet lenses are needed to achieve the required tunability. Various approaches of adaptive lenses^2^ have been demonstrated including in particular liquid lenses with a wide tuning range and high modulation rates in the kHz range. The refracting interface is typically a free liquid surface^3^,^4^,^5^,^6^,^7^,^8^ or a thin elastomeric membrane covering the liquid. In the former case, electrowetting^9^ is the most popular tool to control the refractive power, in the latter case various types of pressure controllers have been explored^10^,^11^,^12^,^13^,^14^. Independent of these differences, fluid pressure is the only control parameter. It allows for manipulating the global curvature of the lens but not the details of its shape. With the exception of a few recent approaches based on deformable polymeric lenses^15^, primary optical aberrations such as spherical aberration are not controlled and remain implicitly determined by the physical laws of capillarity and elasticity.

For interfaces between a conductive and non-conductive liquid, however, it is possible to exert additional stresses on the interface by applying electric fields^16^,^17^. The resulting elliptic distortions of drops were first reported by Zeleny^18^ later analyzed in detail by Taylor^19^, and more recently exploited to tailor the shape of UV curable microlenses^20^,^21^,^22^. Recent studies in the context of electrowetting demonstrate that rather complex liquid surfaces profiles can be generated and tuned in a perfectly reversible manner depending on the local distribution of the electric field^8^,^23^,^24^,^25^. In this report, we exploit these ideas and introduce a novel concept of adaptive liquid micro-lenses, in which we use hydrostatic pressure and Maxwell stress as two separate control parameters for tuning the shape of the interface between two immiscible liquids, denoted for simplicity as oil and water (see methods for details). This approach enables independent tuning of paraxial curvature and ellipticity of the oil-water interface and hence independent control of focal length and spherical aberration.

## Results

**Figure 1:**
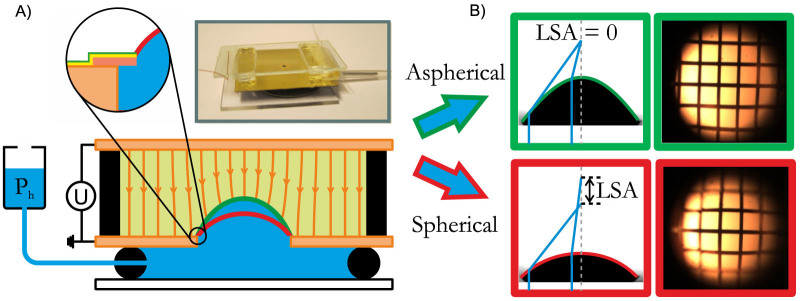
(A) Schematic of the device. The curvature of oil (yellow)-water (blue) interface in the central aperture is regulated by a hydrostatic head Δ*P_h_* through a needle inserted in the O-ring, and a voltage *U* applied between the aperture plate and top electrode. Inset: detail of aperture design to guarantee contact line pinning. Top inset: photograph of the actual device and its connections. (B) interface profiles of a perfect aspherical lens with zero LSA when the correct voltage is applied (top) and of a spherical lens at zero voltage (bottom) along with optical images of a square grid demonstrating the suppression of aberrations.

Our lens consists of three parallel glass plates held at fixed distances by spacers. The middle plate contains a circular aperture with a diameter of 1 mm, the upper plate is covered by a homogeneous transparent electrode. The space between the two lower plates is filled with water, the one between the upper plates is oil-filled (Fig. 1). The oil-water interface is pinned to the edge of the aperture (see methods for details). The aqueous phase is electrically grounded and mechanically coupled to a hydrostatic head that allows for tuning the applied pressure. The oil phase is kept at ambient pressure.

**Figure 2:**
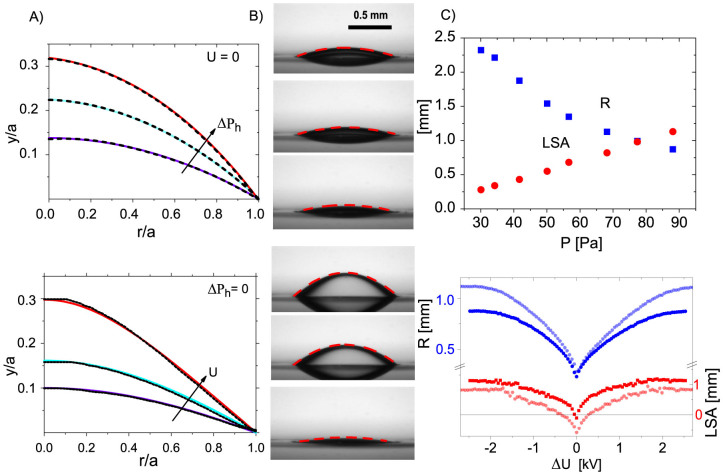
(A) Experimental (black) versus numerical (color) spherical interface profiles at zero voltage and increasing hydrostatic pressure 30 Pa, 68 Pa and 88 Pa (top left) and for aspherical interfaces at zero hydrostatic pressure for increasing voltage 1400 V, 1600 V and 1700 V (bottom left). (B) Middle column shows corresponding interface images and their extracted interface fits (dotted red lines). (C) Top: variation of paraxial radius of curvature R (blue) and LSA (red), for spherical profiles at zero voltage at a hydrostatic pressure of 30, 68 and 88 Pa, respectively. Bottom: R and LSA for aspherical lenses as a function of ramp voltage Δ*U* = *U_max_* − *U* where *U_max_* is the maximum voltage for each ramp. Light symbols correspond to Δ*P_h_* = 68 Pa, dark symbols to Δ*P_h_* = 88 Pa.

At zero voltage, the oil-water interface assumes a spherical shape with a curvature that is controlled by the applied hydrostatic pressure. For applied differential pressures of Δ*P_h_* = 30…88 Pa the radius of curvature of the interface can be tuned between *R* = 2 mm and 0.8 mm, following the Young-Laplace law Δ*P* = 2*γκ*, where *γ* is the interfacial tension and *κ* is the mean curvature of the interface, i.e. 1/*R* for a sphere. This corresponds to (paraxial) focal lengths *f* = *R*/(*n* − 1) = 20…8 mm where *n* = *n_aq_/n_oil_* = 1.10 is the ratio of the refractive index of the two liquids (Fig. 2 top). (Concave interface shapes resulting in divergent lenses can also be produced by applying a negative hydrostatic pressure. They remain stable as long as the contact line remains pinned to the edge following Gibbs' criterion^26^. For the remainder of this communication we will focus on convex lenses.)

Purely pressure-controlled liquid lenses display positive spherical aberration because *R* has one fixed value independent of the distance from the optical axis. While paraxial rays are focused at the nominal focal distance given above, rays entering the lens farther away from the axis are focused closer to the lens. The difference in focal point between paraxial and marginal beams is known as longitudinal spherical aberration (LSA). It leads to defocussing for off-axis rays. Next to defocussing, the spherical shape also induces a characteristic barrel distortion as easily visible in images of rectangular grids as test structures taken with spherical lenses (Fig. 1b). To suppress spherical aberration the local curvature of the oil-water interface must decrease with increasing distance from the optical axis. Such surface profiles can be created if we keep Δ*P_h_* fixed in our device and gradually increase *U* (Fig. 2 bottom). In this situation, the electric field *E* pulls the oil-water interface upward until the electric force is balanced by an increased Laplace pressure due to an increased local curvature. Hence, for paraxial rays *f* decreases. However, because the distance between the oil-water interface and the top electrode is smaller on the optical axis than elsewhere, *E* decreases with increasing distance from the optical axis. The curvature of the lens decreases along with it, as required for an aspherical lens. Quantitatively, the equilibrium shape of the lens is determined by the local stress balance at the oil-water interface 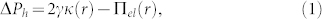
where 
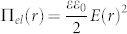
 is the electric Maxwell stress. (*εε*_0_: dielectric permittivity of the oil). Self-consistent numerical calculations of field distribution and interface shape (see methods) show a decrease of the paraxial focal length with increasing *U* (Fig. 2a bottom) Simultaneously, the LSA decreases, in quantitative agreement with the experiments (Fig. 2c bottom). Thanks to the fact that the edge of the oil-water interface is perfectly pinned to the edge of the aperture, deformations of the lens do not involve any contact line motion. As a consequence, the lens can be tuned very smoothly without appreciable hysteresis (see supplementary information). At a certain critical voltage *U*_c_, the lens is perfectly aspherical, as indicated by the vanishing LSA. For even higher voltages, the LSA assumes negative values. The shape of a perfectly aspherical lens is given by a hyperbola 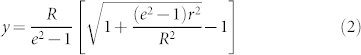
with an eccentricity *e* = *n* = 1.10. R is the radius at the apex, which determines the focal length. Both the calculated and the experimental profile fulfill this criterion for *U* = *U_c_* to an excellent degree of accuracy. This perfect lens shape is readily obtained for the present geometry of a simple flat electrode placed at some distance from the lens (Fig. 1a). Optical images of our test grid indeed demonstrate near perfect focus across the entire field of view, including suppression of barrel distortion (Fig. 1b).

**Figure 3:**
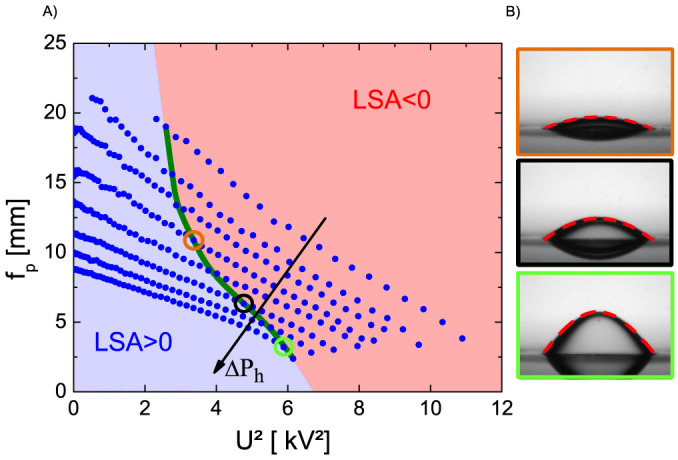
(A) Focal length versus applied voltage squared (blue symbols) as measured for a range hydrostatic pressures increasing from 30 Pa to 88 Pa (top to bottom). Measurement points in the red area showed a negative LSA, while points in the blue area showed a positive LSA. Hence, the green curve represents the interfaces with zero LSA. (B) Interface images with extracted fits of lenses at zero LSA for Δ*P_h_* = 50, 68 and 88 Pa corresponding to the three encircled points in the left panel.

Applying a suitable voltage thus enables perfect asphericity. Yet, the simultaneous variation of the focal length limits the versatility of using electric fields as only control parameter. To achieve fully independent control of *f* and LSA, both pressure and voltage are varied simultaneously. By varying U from 0 V to 3.3 kV and Δ*P_h_* from 30 Pa to 88 Pa, we can cover a range of focal lengths and spherical aberrations from *f* = 20…8 mm and *LSA* = −1.79 …+ 1.13 mm respectively, as shown in Fig. 3. In particular, perfectly aspherical conditions can be maintained over the full range of focal lengths by adjusting *U* and Δ*P_h_* accordingly. (Corresponding surface profiles along with conical fits are shown in the supplementary information.)

## Discussion

The approach presented here is not limited to the suppression of spherical aberration under quasi static conditions. First of all, much more flexible – almost arbitrary – distributions of electric fields and hence lens shapes can be generated if the electrode on the top surfaces is divided into individually addressable segments as routinely used in electrowetting experiments and display technology^27^. It is straightforward to combine our approach with an electrically addressable pressure controller to replace the hydrostatic head used in the present experiments. Various approaches including electrowetting^3^,^28^ have been used to demonstrate switching speeds well above video rate. Particularly high switching rates in the kHz range should be possible for smaller lens apertures as used e.g. in micro-lens arrays^8^. We anticipate that our approach will pave the way for a new generation of adaptive optofluidic devices with superior optical performance.

## Methods

The device consists of three glass plates (fig. 1). Top and bottom plate are covered with transparent electrodes made of Indium-Tin-Oxide (ITO). The bottom and the middle one are separated by an O-ring with a diameter of 1.4 cm and a thickness of 3 mm, respectively. A hole with a diameter of 1.2 mm is drilled into the middle plate on the optical axis. To guarantee a well-defined aperture with smooth edges, a small disc made from Cu (Supplied by Agar scientific) with a thickness 50 μm and a central hole with a diameter of 1 mm is glued onto the middle plate aligned with the hole. The space below the middle plate is filled with an aqueous solution of CsI (type:202134; Sigma Aldrich) with a refractive index of *n_aq_* = 1.55 and density *ρ_aq_* = 1.05 gm/ml. The space between the middle and the top plate is filled with silicone oil (type:378348; Sigma Aldrich) with a refractive index *n_oil_* = 1.41 and density *ρ_oil_* = 0.95 gm/ml. (Combining this density mismatch and the dimensions of the device results in a Bond number *Bo* = Δ*ρgR*^2^/γ = 0.006 ≪ 1, which indicates that gravity is negligible in our experiments.). Prior to assembling the device a thin Au layer is thermally evaporated onto the middle plate with the Cu disk to make the upper side of the middle plate conductive. To guarantee perfect pinning of the oil-water interface to the edge of the aperture, a hydrophobic thiol coating is applied to the Au layer by immersing it into a dilute solution of 1-Dodecanethiol in 99% ethanol for 24 hrs.

### Measurement of focal length and LSA

Focal length and LSA are calculated with a ray-tracing code written in MATLAB using the measured interface profile and the refractive indices of all materials as input. For a ray of light propagating towards the interface parallel to the optical axis at a radial distance r, the refracted ray from the interface is calculated using Snell's law. For this refracted ray the position where it crosses the optical axis has been calculated as a function of the distance r. The spread in these crossing positions between paraxial and marginal rays defines the LSA.

### Drop profile extraction and fits

Drop profiles are extracted from the recorded images by taking the gradient of the intensity variation across the oil-water interface in each pixel row. The intensity across the interface is sigmoidal in nature, while its gradient is Gaussian. Drop profile is obtained by connecting the peaks of the fitted Gaussian curves at each scan line. The extracted profiles are subsequently fitted with the standard conic section equation, Eq. (2) to extract the radius of curvature *R* at the apex and the eccentricity *e*.

### Simulation versus experimental profiles

Numerical profiles in Fig. 2a (bottom) are calculated by a self-consistent calculation of the electric field distribution and the shape of the oil-water interface using a finite element method as implemented in the commercial software package COMSOL MULTIPHYSICS using an axisymmetric coordinate system. The conductive water phase as well as the gold coated middle plate are kept at zero potential. The flat top electrode is kept at a the fixed applied potential^25^ (see Fig. 1a). The electric field distribution in the oil phase, with relative dielectric permittivity *ε*, is obtained by solving the Laplace equation. Numerical profiles are obtained from the local balance of the Maxwell stress and Laplace pressure along the oil-water interface (Eq. 1). Interface equilibration is tracked using a moving mesh algorithm (Arbitrary Lagrangian Eulerian; ALE). 

## Author Contributions

K.M., C.M. and B.C. performed the experiments. K.M., C.M. and G.M. assissted in sample preparation. K.M., B.C. and D.V.D.E. analyzed the data. K.M. and D.V.D.E. took care of the theoretical description. I.R. and J.M.O. carried out simulations. and F.M. conceived the original idea and wrote the manuscript.

## Supplementary Material

Supplementary Information Movie S1

Supplementary Information Figure S1

## References

[b1] Zappe H. Fundamentals of Micro-Optics. (Cambridge University Press, 2010).

[b2] Reichelt S. & Zappe H. Design of spherically corrected, achromatic variable-focus liquid lenses. Opt Express 15, 14146–14154 (2007). 19550687 10.1364/oe.15.014146

[b3] Berge B. & Peseux J. Variable focal lens controlled by an external voltage: An application of electrowetting. Eur Phys J E 3, 159–163 (2000).

[b4] Krupenkin T., Yang S. & Mach P. Tunable liquid microlens. Appl Phys Lett 82, 316–318 (2003).

[b5] Kuiper S. & Hendriks B. H. W. Variable-focus liquid lens for miniature cameras. Appl Phys Lett 85, 1128–1130 (2004).

[b6] Dong L., Agarwal A. K., Beebe D. J. & Jiang H. R. Adaptive liquid microlenses activated by stimuli-responsive hydrogels. Nature 442, 551–554 (2006). 16885981 10.1038/nature05024

[b7] Lopez C. A. & Hirsa A. H. Fast focusing using a pinned-contact oscillating liquid lens. Nat Photonics 2, 610–613 (2008).

[b8] Murade C. U., van der Ende D. & Mugele F. High speed adaptive liquid microlens array. Opt Express 20, 18180–18187 (2012). 23038366 10.1364/OE.20.018180

[b9] Mugele F. & Baret J. C. Electrowetting: From basics to applications. J Phys-Condens Mat 17, R705–R774 (2005).

[b10] Kyle C., Fainman Y. & Groisman A. Pneumatically actuated adaptive lenses with millisecond response time. Appl Phys Lett 91 (2007).

[b11] Shi L., Shi J., McManamon P. F. & Bos P. J. Design considerations for high efficiency liquid crystal decentered microlens arrays for steering light. Appl Optics 49, 409–421 (2010). 10.1364/AO.49.000409 20090805

[b12] Zhu D. F., Lo C. W., Li C. H. & Jiang H. R. Hydrogel-Based Tunable-Focus Liquid Microlens Array With Fast Response Time. J Microelectromech S 21, 1146–1155 (2012).

[b13] Ren H. W., Xianyu H. Q., Xu S. & Wu S. T. Adaptive dielectric liquid lens. Opt Express 16, 14954–14960 (2008). 18795032 10.1364/oe.16.014954

[b14] Dai H. T., Liu Y. J., Sun X. W. & Luo D. A negative-positive tunable liquid-crystal microlens array by printing. Opt Express 17, 4317–4323 (2009). 19293856 10.1364/oe.17.004317

[b15] Ren H. W., Xu S. & Wu S. T. Deformable liquid droplets for optical beam control. Opt Express 18, 11904–11910 (2010). 20589052 10.1364/OE.18.011904

[b16] Brown C. V., Wells G. G., Newton M. I. & McHale G. Voltage-programmable liquid optical interface. Nat Photonics 3, 403–405 (2009).

[b17] Hou L., Smith N. R. & Heikenfeld J. Electrowetting manipulation of any optical film. Appl Phys Lett 90 (2007).

[b18] Zeleny J. Instability of Electrified Liquid Surfaces. Physical Review 10, 1–6 (1917).

[b19] Taylor G. Disintegration of Water Drops in Electric Field. Proc R Soc Lon Ser-A 280, 383 (1964).

[b20] Zhan Z. X., Wang K. Y., Yao H. T. & Cao Z. L. Fabrication and characterization of aspherical lens manipulated by electrostatic field. Appl Optics 48, 4375–4380 (2009). 10.1364/ao.48.004375 19649041

[b21] O'Neill F. T., Owen G. & Sheridan J. T. Alteration of the profile of ink-jet-deposited UV-cured lenses using applied electric fields. Optik 116, 158–164 (2005).

[b22] Chen C. W. & Tseng F. G. Tunable micro-aspherical lens manipulated by 2D electrostatic forces. Transducers '05, Digest of Technical Papers, *Vols 1 and 2*, 376–379 (2005).

[b23] Buehrle J., Herminghaus S. & Mugele F. Interface profiles near three-phase contact lines in electric fields. Phys Rev Lett 91 (2003). 10.1103/PhysRevLett.91.086101 14525260

[b24] Manukyan G., Oh J. M., van den Ende D., Lammertink R. G. H. & Mugele F. Electrical Switching of Wetting States on Superhydrophobic Surfaces: A Route Towards Reversible Cassie-to-Wenzel Transitions. Phys Rev Lett 106 (2011). 10.1103/PhysRevLett.106.014501 21231746

[b25] Oh J. M., Manukyan G., van den Ende D. & Mugele F. Electric-field-driven instabilities on superhydrophobic surfaces. Epl-Europhys Lett 93 (2011).

[b26] Rathgen H., Sugiyama K., Ohl C. D., Lohse D. & Mugele F. Nanometer-resolved collective micromeniscus oscillations through optical diffraction. Phys Rev Lett 99 (2007). 10.1103/PhysRevLett.99.214501 18233223

[b27] Heikenfeld J. *et al.* Electrofluidic displays using Young-Laplace transposition of brilliant pigment dispersions. Nat Photonics 3, 292–296 (2009).

[b28] Murade C. U., Oh J. M., van den Ende D. & Mugele F. Electrowetting driven optical switch and tunable aperture. Opt Express 19, 15525–15531 (2011). 21934915 10.1364/OE.19.015525

