# Supplementary material for: Optofluidic lens with tunable focal length and asphericity
Source: Sci Rep. 2014 Sep 16;4:6378. doi: 10.1038/srep06378 (PMC4165269; doi:10.1038/srep06378)
Supplement: Supplementary Information — Figure S1 [file srep06378-s2.pdf]

Optofluidic lens with tunable focal length and asphericity

Kartikeya Mishra[1], Chandrashekhar Murade[1], Bruno Carreel[1], Ivo Roghair[2], Jung Min Oh[1], Gor Manukyan[1], Dirk van den Ende[1], and Frieder Mugele[1]

[1]University of Twente – MESA+ institute for Nanotechnology – Physics of Complex Fluids; PO Box 217; 7500 AE Enschede (The Netherlands)

(correspondence should be addressed to F.M. (email: f.mugele@utwente.nl)

**Movie S1**: Side view image of lens shape for $\Delta P_h$ = 88 Pa upon ramping the applied voltage from 0 to 2.48KV and back. Lens attains starting from positive to zero and then to negative values of LSA.

**Figure S1**: Examples of lens profiles with zero spherical aberration.

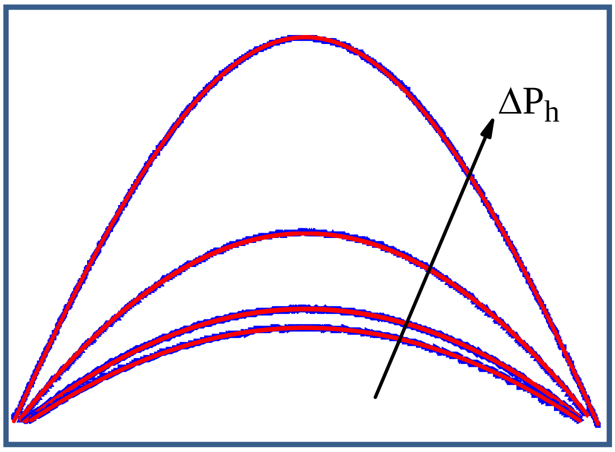


Surface profiles and conical section fits for perfect lenses with zero LSA at increasing values of $\Delta P_h$ as specified in the table below from bottom to top. For all profiles, the fitted value of the ellipticity e is close to the refractive index ratio n=1.10 (see table) with an error of ±0.02.

| | $\Delta P_h$ [Pa] | $f_p$ [mm] | e |
|---|---|---|---|
| 1 | 34 | 15.60 | 1.10 |

| | | | |
|---|---|---|---|
| 2 | 50 | 10.87 | 1.09 |
| 3 | 68 | 6.33 | 1.11 |
| 4 | 88 | 2.38 | 1.11 |

[2]Eindhoven University of Technology, Department of Applied Physics, Mesoscopic Transport Properties Group, P.O. Box 513, 5600MB Eindhoven (The Netherlands)
